# Effect of the duration of antimicrobial exposure on the development of antimicrobial resistance (AMR) for macrolide antibiotics: protocol for a systematic review with a network meta-analysis

**DOI:** 10.1186/s13643-018-0917-0

**Published:** 2018-12-23

**Authors:** Titus H. Divala, Elizabeth L. Corbett, Helen R. Stagg, Marriott Nliwasa, Derek J. Sloan, Neil French, Katherine L. Fielding

**Affiliations:** 1 London School of Hygiene & Tropical Medicine, Keppel Street, Bloomsbury, London, WC1E 7HT UK; 2 Helse Nord Tuberculosis Initiative, University of Malawi College of Medicine, Blantyre, Malawi; 3 Liverpool Wellcome Trust Clinical Research Programme, College of Medicine, University of Malawi, Blantyre, Malawi; 4 Usher Institute for Population Health Sciences and Informatics, University of Edinburgh, Edinburgh, EH8 9AG UK; 5 School of Medicine, University of St Andrews, St Andrews, UK; 6 Institute of Infection & Global Health, University of Liverpool, Liverpool, UK

**Keywords:** Antimicrobial resistance, Network meta-analysis, Macrolides, *Streptococcus pneumoniae*, Carriage, Treatment duration, Treatment failure, Disease recurrence, Resistance mechanisms, Prescriptions

## Abstract

**Background:**

Antimicrobial resistance generates a huge health and economic burden and has the potential to become the leading cause of death globally, but its underlying drivers are yet to be fully described. The association between a microbe’s exposure to antimicrobials and subsequent development of, or selection for, resistance is well documented, as are the exacerbating microbial and human factors. However, the nature and extent of this risk, and how it varies by antimicrobial class and duration of treatment, is poorly defined. The goal of our systematic review and network meta-analysis is to determine the relationship between the duration of antimicrobial exposure and selection for resistance. We will use macrolides as the antimicrobial class of interest and *Streptococcus pneumoniae* carriage as an indicator organism. Our secondary outcomes include duration of symptoms, risk of treatment failure and recurrence, and descriptions of resistance mechanisms.

**Methods:**

We will conduct a systematic review, selecting studies if they are published randomised controlled trials (RCTs) which report the relationship between taking a macrolide for any indication and incidence of resistant *Streptococcus pneumoniae* in patients of any age group. We will use a predefined search strategy to identify studies meeting these eligibility criteria in MEDLINE, Embase, Global Health and the Cochrane Central Register of RCTs. Two authors will independently screen titles and abstracts, review the full texts and undertake data extraction. We will use the Cochrane Collaboration’s tool to assess the quality of included RCTs. If feasible, we will perform pair-wise meta-analysis modelling to determine the relationship between the duration of macrolide treatment and development of macrolide resistant *Streptococcus pneumoniae.* If the identified studies meet the assumptions for a network meta-analysis (NMA), we will additionally model this relationship using indirect comparisons. Our protocol utilises reporting guidance by Preferred Reporting Items for Systematic Reviews and Meta-analyses (PRISMA) and the extensions for protocols (PRISMA-P) and network meta-analyses (PRISMA for NMA). Our review will also report to these standards.

**Discussion:**

Establishing the relationship between the duration of antimicrobial exposure and development of, or selection for, resistance will inform the design of antimicrobial prescriptions, treatment guidelines and the behaviour of both physicians and patients. This work will therefore be a strong contribution towards the full realisation of current antimicrobial resistance stewardship strategies.

**Systematic review registration:**

PROSPERO CRD42018089275

**Electronic supplementary material:**

The online version of this article (10.1186/s13643-018-0917-0) contains supplementary material, which is available to authorized users.

## Background

Antimicrobials—organic or synthetic molecules with cytotoxic or cytostatic abilities against microbes—are one of the greatest medical discoveries [1]. Unfortunately, their usefulness is limited by the inherent genetic capacity of microbes to rapidly develop, transfer and acquire resistance-causing mutations [2, 3]. Unnecessary prescription in medical settings, as well as extensive agricultural use, contributes substantially to overall antibiotic drug pressure globally [4–6]. The current era is characterised by sharply declining investment from the pharmaceutical industry in the development of effective new antimicrobials; far fewer new compounds are developed annually now than during the 1990s [7].

In 2016, antimicrobial resistance became one of only four health topics ever to be discussed at the United Nations General Assembly [8], reflecting its huge health and economic burden [9, 10]. Drug resistance is projected to become the leading cause of death by 2050 [11].

The development of antimicrobial resistance is, to some extent, inevitable. Billions of doses of antibiotics are taken globally each year. Each human hosts a microbiome of approximately 3.8 × 10^13^ bacteria [12], and there is spontaneous (i.e. unselected) drug-resistance within this microbiome at a frequency as high as 10^−4^ mutations, depending upon the type of antimicrobial [13–15]. Under drug pressure, resistance can then be amplified and transmitted through a variety of mechanisms [2]. Despite these risks, use of single-drug regimens remains standard practice for many conditions, because they are often sufficient to cure the patient and they reduce immediate costs and adverse events.

There is unambiguous evidence that the development of, or selection for, resistance occurs following antimicrobial exposure [4, 16–18]. The duration of treatment that is necessary for the development of, or selection for, resistance is poorly defined, however. This may differ by the resistance mechanism; for example, the unmasking of any resistant organisms generated during previous treatment periods may rapidly occur following a brief exposure to antimicrobials, while de novo generation may require longer durations. Better quantification of this relationship will inform prescription design, guidelines and behaviour, all of which are key factors in effective antimicrobial resistance control strategies [19].

To explore this relationship and derive high-quality evidence, it is necessary to choose an antimicrobial or class of antimicrobials with which patients are treated, an indicator organism in which drug resistance develops and a uniform method for assessing resistance. Macrolides are one of the most prescribed antimicrobials in clinical practice [20, 21], which act by inhibiting bacterial protein synthesis [22]. *Streptococcus pneumoniae*, a major aetiology of clinical illness [23–26], also harmlessly colonises the upper respiratory tract, creating a window for the assessment of circulating serotypes and resistance patterns. *Streptococcus pneumoniae* is also a popular indicator bacteria in randomised controlled trials (RCTs) as globally accepted laboratory procedures for its detection exist [27–29] and colonisation is more common than invasive pneumococcal disease [30–32]. The existence of internationally accepted laboratory standards presents opportunities for between-study comparisons.

Our aim, therefore, is to conduct a systematic review using data from published RCTs to determine the relationship between the duration of antimicrobial exposure and the development of, or selection for, resistance using carriage of *Streptococcus pneumoniae* as an indicator organism. We will evaluate studies involving healthy individuals or patients with any illness treated with macrolide antimicrobials in whom the development of, or selection for, antimicrobial resistance was assessed using *Streptococcus pneumoniae* carriage. We have opted to use network meta-analysis (NMA) as the preferred evidence synthesis method because RCTs with head-to-head comparisons of different durations of macrolides will likely be too rare for a meaningful pair-wise meta-analyses. NMAs allow the use of both direct and indirect evidence and are hence the most efficient method for making inferences.

Our primary objective is to determine whether the risk of developing macrolide resistance increases with the duration of macrolide exposure using upper respiratory tract carriage of *Streptococcus pneumoniae* as indicator organism. Our secondary objectives include exploring the association between the duration of macrolide treatment and (1) symptom duration, (2) treatment failure and (3) disease recurrence.

## Methods

### Protocol and registration

The protocol for this planned systematic review is registered with the International Prospective Register of Systematic Reviews (PROSPERO), CRD42018089275.

### Eligibility criteria

We will include studies that fulfil the following criteria:

1. *Population*: healthy individuals or patients of any illness or age, treated with macrolide antimicrobials. We will record participant characteristics, including age, sex and the indication for treatment.
2. *Interventions*: Any macrolide antimicrobial being given as monotherapy, via any route, for respiratory infections. We are interested in the impact of macrolide treatment on antimicrobial resistance in *Streptococcus pneumoniae* carriage. We will record the specific macrolide, dose and duration reported in each study. Registered macrolides include azithromycin, clarithromycin, erythromycin, fidaxomicin, telithromycin, carbomycin a, josamycin, kitasamycin, midecamycin, midecamycin acetate, oleandomycin, solithromycin, spiramycin, troleandomycin, tylosin, tylocine and roxithromycin.
3. *Comparators*: Other macrolides, other antimicrobials, placebo, or no treatment. Macrolides are commonly used for respiratory tract infections, and amoxicillin and doxycycline are expected to be the most frequent non-macrolide comparators.
4. *Outcomes*: The primary outcome will be the incidence/risk of macrolide resistance in *Streptococcus pneumoniae* carriage among individuals in whom this did not exist before commencing macrolide treatment. Macrolide resistance in *Streptococcus pneumoniae* results from either ribosomal dimethylation by an enzyme encoded by erm(B), efflux by a two-component efflux pump encoded by mef (E)/mel(msr(D)), or mutations of the ribosomal target site of macrolides [33]. We will include studies that utilised any established laboratory method to demonstrate evidence of macrolide resistance.

Our secondary outcomes include the duration of symptoms (number of symptomatic days from commencement of therapy), risk of treatment failure (persistence of symptoms after completing a dosage of antimicrobials) and disease recurrence (re-emergence of disease within 4 weeks of the resolution of previous symptoms).

5. *Study design*: RCTs. Restricting to RCTs will minimise confounding.
6. *Language and time limitations*: We will include studies published in any language and on any date. For articles in languages other than English that are eligible for full-text review, we will seek assistance from a native speaker who has been trained in data extraction using an article published in English.

### Anticipated network geometry

**Fig. 1:**
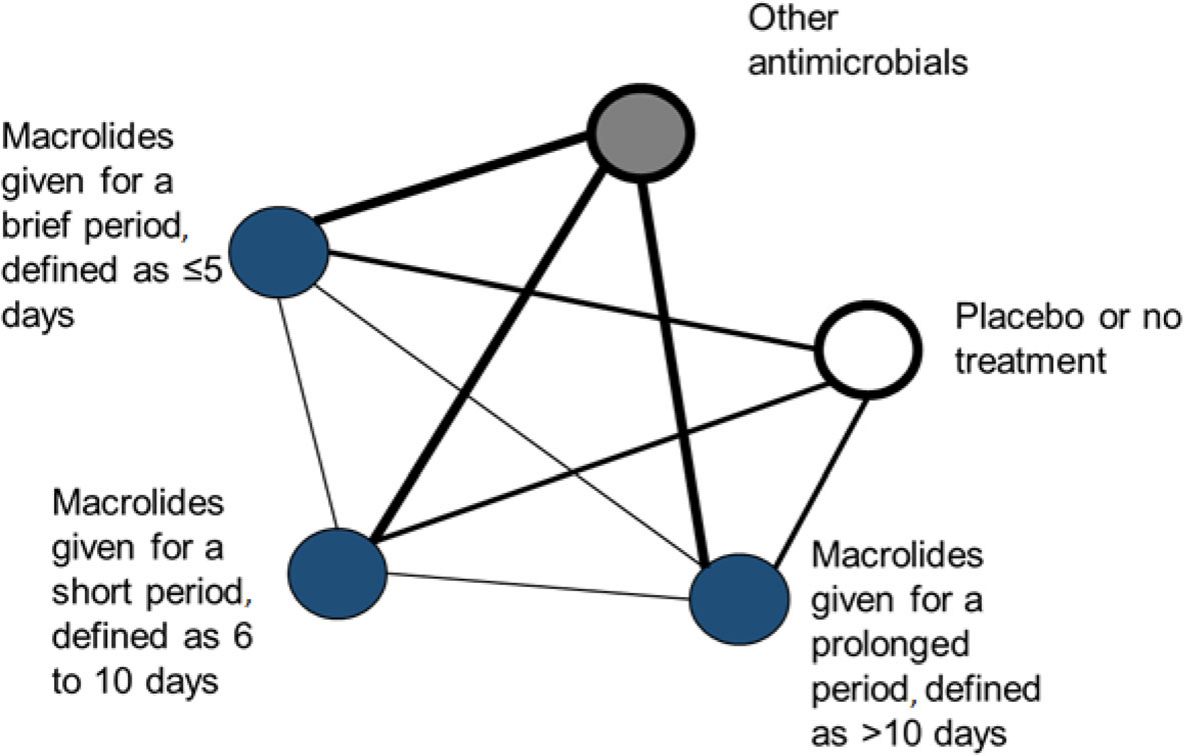
Hypothetical network of anticipated randomised controlled trial data for the effect of macrolide treatment duration on the development of antimicrobial resistance. Each treatment group is a node. The lines joining nodes, termed edges, will be drawn to thickness that graphically represents the amount of direct evidence: the number of comparisons that we expect to find between a particular pair of nodes

In NMA geometry, competing interventions are represented by points termed nodes. In this case, nodes are the duration of exposure to any macrolide used in the included RCTs (Fig. 1). We will classify treatment duration as brief if it is ≤ 5 days, short if it is 6 to 10 days, and prolonged if it is > 10 days. These durations are based on an understanding of the current clinical use of macrolides, which are dosed for up to 5 days for community acquired pneumonia, up to 10 days for severe pneumonia, and for prolonged periods of time for inflammatory respiratory illnesses such as cystic fibrosis and non-cystic fibrosis bronchiectasis. Within the network, the lines joining nodes are termed edges and are drawn to a thickness that graphically represents the anticipated amount of evidence or number of comparisons that we expect to find between the particular nodes. For example, it is likely that more RCTs will compare a macrolide to a control than to another macrolide of a different duration. NMAs will allow us to compare different durations of macrolide exposure (brief, short and prolonged) by computing indirect comparisons, provided that any patient meeting our eligibility criteria would, theoretically, have been equally likely to be randomised to any of the interventions of the studies included in the network.

### Information sources and search strategy

We will search for studies that meet the eligibility criteria in MEDLINE, Embase, Global Health, Web of Science and Cochrane Central Register of Controlled Trials (CENTRAL: The Cochrane Library). MEDLINE, Embase and Global Health will be searched using the Ovid platform. Papers will not be excluded on the basis of the language of publication and time frame in which they were published. We will only include data from peer-reviewed papers in order to ensure scientific quality.

In Table 1, we present our search strategy for MEDLINE, which we will adapt for the other databases. This strategy was reviewed by an information retrieval expert from the London School of Hygiene & Tropical Medicine (LSHTM) library. After running the search, we will export results to Endnote X8 and remove all duplicates. We will also include any relevant articles identified from the reference lists of included articles.

**Table 1:** Search strategy for MEDLINE using Ovid platform

Theme	Line number	Searches
Antimicrobial resistance	1.	drug resista* or exp drug resistance, microbial/
	2.	bacterial resistan*.ti,ab.
	3.	antimicrobial resistan*.ti,ab.
	4.	1 or 2 or 3
Macrolides	5.	MACROLIDES/
	6.	(Azithromycin or Clarithromycin or Erythromycin or Fidaxomicin or Telithromycin or Carbomycin A or Josamycin or Kitasamycin or Midecamycin or midecamycin acetate or Oleandomycin or Solithromycin or Spiramycin or Troleandomycin or Tylosin or tylocine or Roxithromycin).ti,ab.
	7.	5 or 6
Antimicrobial resistance studies that use macrolides (*any design*)	8.	4 and 7
MEDLINE filter for clinical trials	9.	randomised controlled trial.pt.
	10.	controlled clinical trial.pt.
	11.	randomised.ab.
	12.	placebo.ab.
	13.	drug therapy.fs.
	14	randomly.ab.
	15.	trial.ab.
	16.	groups.ab.
	17.	arms.ab.
	18.	9 or 10 or 11 or 12 or 13 or 14 or 15 or 16 or 17
	19.	exp animals/ not humans.sh.
	20.	19 not 18
Antimicrobial resistance studies that use macrolides in clinical trials	21.	8 and 20

### Study selection

Investigator THD will implement the search strategy, and then investigators THD and MN will screen the titles and abstracts of resulting papers against the eligibility criteria. THD and MN will independently assess the full texts of the included papers for eligibility using the above criteria. The main reason for non-inclusion at the full-text stage will be documented. Investigator KF will resolve any disagreements.

### Data extraction

Publication information will be exported from Endnote into a standardised extraction form in Microsoft Excel Data will be extracted into (Additional file 1). This form is currently in draft format; it will be finalised among the study team once it has been trialled by two people extracting the same five papers. After finalisation of the form, two team members will extract the data independently. Discrepancies will initially be discussed and resolved between the two team members, with a third team member available to resolve disputes. Multiple publications arising from the same study will be combined. Where data gaps are present, the original study authors will be contacted. Once the extraction phase is complete, data will be exported into the analysis software.

#### Data for assessing methodological comparability of trials

In addition to the data necessary for outcome evaluation, we will extract information on any interventions, or study or population characteristics that may act as effect modifiers, as is necessary for the assessment of the assumptions of the NMA. These are:

1. Methods: study design, randomisation (individual or cluster), total duration of study, number of study centres and location, study setting, withdrawals and date of study.
2. Participants: age, number, setting, eligibility criteria and baseline antimicrobial resistance (AMR).
3. Interventions: indication of treatment, dose of both the macrolide and control interventions and duration of treatment.
4. Outcomes: authors’ primary and secondary outcomes, timing for assessing AMR in relation to the treatment administration schedule and participant adherence levels. We will attempt to extract outcome data per study arm, as opposed to summary effects.
5. Additional factors: trial sponsorship, trial funding and important conflicts of interest reported by the authors.

#### Data from cross-over and cluster randomised trials

The units of analysis in cross-over and cluster randomised trials (CRTs) need special considerations before meta-analysis is undertaken in order to address carry-over effects and clustering, respectively. For cross-over studies we will only extract data from the first period, while for CRTs, we will extract data that accounts for the clustering.

### Risk of bias assessment

We will conduct a risk of bias assessment at the level of the study. We will use the revised Cochrane risk-of-bias tool for randomised trials (RoB 2.0), the recommended method for assessing experimental studies [34]. The risk of bias assessment tool interrogates various aspects of selection and information bias. It involves assessing how the allocation sequence was generated, how it was concealed, if blinding was done, how outcomes were ascertained, the quality of follow up, and whether there was selective outcome reporting. The risk of bias assessment will be done independently by two reviewers and disagreements resolved by discussion or by third reviewer.

### Data analysis

#### Guiding counterfactual model

**Fig. 2:**
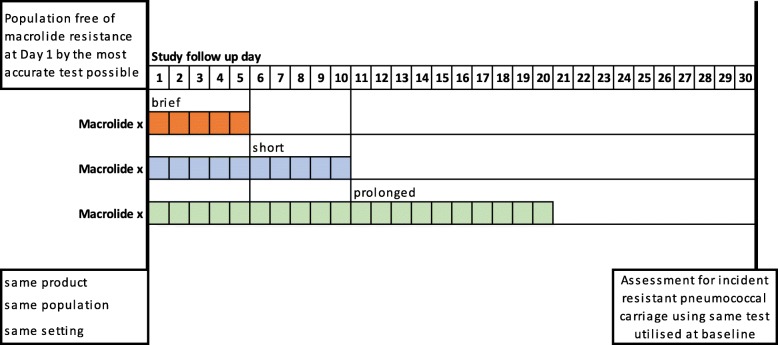
Counterfactual framework guiding analysis for the primary outcome of the systematic review

Our analysis will strive, as far as possible, to mimic the counterfactual framework presented in Fig. 2. The ideal study for addressing the primary outcome is one that recruits AMR-free participants of similar demographics, randomises them (1:1:1) to receiving any of the three durations of the same macrolide antimicrobial (brief, short and prolonged), and then follows them for the same duration before assessing for AMR using the same technique. Restricting to the same type of macrolide antimicrobial would limit the impact of the inherent differences in the intervention itself. For example, within the macrolide class, the drugs have different bioavailability and half-lives; this may impact the development of, or selection for, resistance. Additionally, different dosing, routes of administration, and strength of activity against *S. pneumoniae* are other sources of variability. Furthermore, an optimal study would assess outcomes in each arm at the same time relative to the end of treatment (e.g. 1 day post-treatment), as macrolide AMR has been shown to decrease with time from last date of treatment. The use of the same technique would ensure comparability of results between arms.

While ideal, achieving all these factors in a real-life systematic review is unlikely. Our final statistical analysis plan will therefore be a calculated trade-off of these ideal conditions.

#### Study and network characteristics

Data will initially be analysed using descriptive statistics, including all the variables described above, in addition to reporting the comparisons performed in each study, indications for antimicrobial therapy, participant characteristics, study setting, and methodological approaches.

We will prepare a network diagram (similar to the hypothetical diagram shown in Fig. 1) in which the size of the nodes reflect the total number of patients randomised to each intervention, the thickness of edges is proportional to the number of direct comparisons, and the colour of each edge will represent the risk of bias. We will use a contribution matrix to understand and rank the influence of various comparisons in the network on the final summary data [35, 36].

#### Pair-wise and network meta-analysis

If sufficiently methodologically homogeneous studies are identified, we will perform pair-wise meta-analysis for the primary outcome using either fixed effects or random effects modelling approaches, depending on the extent of heterogeneity. We will assess the extent of heterogeneity using the Cochran *Q*^2^ and *I*^2^ statistics. We will convey the extent of heterogeneity visually using a forest plot.

Next, we will assess whether the identified studies meet the assumptions for a NMA. Apart from having reasonably homogeneous methodologies, the key assumption for ensuring validity of inferences drawn from indirect comparisons within a network is transitivity; the balance of the distributions of patient and study characteristics across studies. Initially, we will determine if this assumption is fulfilled by conducting a qualitative review of the RCT characteristics described earlier (‘Data extraction’ section).

For the subset of eligible studies in which the transitivity assumption holds, we will assume that each of their patients were equally likely to be randomised to any of the antimicrobial agents and treatment durations being investigated, thus establishing the basis for the indirect comparisons. Fixed and random effects NMAs will then be used to synthesise all the evidence for the primary outcome and to rank included treatments. To identify the appropriate model between fixed and random effects NMAs for our data, we will use the deviance information criteria (DIC) to assess their goodness-of-fit. The summary effect measures for all pairwise comparisons will be presented in a league table. We will rank the risk of AMR with various treatments using the surface under the cumulative ranking curve (SUCRA) and mean ranks [37].

Consistency within the network—the agreement between direct and indirect evidence—will be assessed within each loop of evidence using loop-specific approach [38] and by employing a global method for evaluating the whole network [39]. We will also estimate the *I*^2^ for network heterogeneity and inconsistency [40, 41], but we will exercise caution when interpreting the results, considering the well-established limitations in power [42]. We will use funnel plot to assess for publication bias.

### Additional analyses

We will perform subgroup, meta-regression and sensitivity NMA analyses. The subgroup analyses will involve running the NMA model stratified by study-level characteristics, i.e. (1) the age groups of participants, (2) country in which the study was conducted, (3) treatment indications, (4) macrolide type and (5) publication calendar period. The meta-regression will include the study-level covariates described earlier (‘Data for assessing methodological comparability of trials’ section), in order to reduce heterogeneity. We will initially add the covariates to the NMA individually, retaining those that have a meaningful impact on the DIC and considering combinations of factors after the initial individual-level assessment. Should we identify additional relevant characteristics during data extraction or analysis for both the subgroup analyses and meta-regression, we will identify such analysis (in our publication) as post hoc. In sensitivity analyses, we will perform the NMA with and without studies that have high risk of bias.

#### Model implementation

We will perform our analyses and report treatment effects on both relative and absolute difference scales, stating odds ratios (ORs), risk differences (RDs) and respective 95% credible intervals (95%CrI) for all comparisons. We will model using OpenBUGS [43] and Stata release 15 (StataCorp, College Station, TX, USA). We will use binomial likelihoods with uninformative prior distributions for our Bayesian modelling. The Brooks-Gelman-Rubin diagnostic will be utilised to assess for model convergence [44, 45]. We will primarily use the mvmeta command [46] in Stata to assess inconsistency and to produce network graphs.

### Credibility of the evidence

The credibility of the evidence will be evaluated with respect to its limitations, indirectness, inconsistency, imprecision and publication bias using the approach recommended by the Grade of Recommendation, Assessment, Development and Evaluation (GRADE) system [47, 48].

### Dissemination of results

We will present the results of our analyses in a peer-reviewed manuscript using the reporting guidance by the Preferred Reporting Items for Systematic Reviews and Meta-analyses (PRISMA) [49] and the PRISMA Network Meta-Analysis extension statement [50]. This work will also form part of a PhD thesis for THD, which he will submit to the LSHTM.

## Discussion

Our systematic review will use published RCTs of macrolide antimicrobials to establish the relationship between the duration of antimicrobial exposure and the development of, or selection for, resistance using upper respiratory tract carriage of *Streptococcus pneumoniae,* isolated from patients with respiratory symptoms, as indicator organism. This will inform the design of antimicrobial prescriptions, treatment guidelines and the behaviour of both physicians and patients. This work therefore will therefore be an important contribution towards the realisation of current antimicrobial resistance control strategies [19].

Where possible, through our secondary objectives, we will attempt to describe the clinical outcomes associated with different macrolide durations. Our results on these outcomes will form the basis for future, detailed, research.

The strengths of our review include publication of the full protocol with PROSPERO and in this peer-reviewed article, with detailed methodology laid out a priori. The internal validity of our review is safeguarded by our restriction of the study type to RCTs. The quality and transparency of our work are ensured by our adherence to both PRISMA and PRISMA NMA guidelines.

The conclusions of our NMA will be weakened if direct comparisons are rare, leading to an overreliance on indirect comparisons. Heterogeneity may be introduced by our broad participant population (all ages, any indication of treatment), global coverage (any setting) and unlimited study period. We will seek to limit this by adding study-level covariates to the NMA model, if required.

To our knowledge, our review and NMA will be the first attempt to systematically examine the association between the duration of exposure to macrolide antimicrobials and subsequent development of, or selection for, resistant *Streptococcus pneumonia* carriage. Therefore, our review will not only provide direction for AMR stewardship policies, but also guide future AMR research.

## Additional file

Additional file 1: Data extraction form. The draft form includes the risk of bias assessment tool and documents the data which will be extracted from included studies. (DOCX 26 kb)
