# Supplementary material for: Effect of the duration of antimicrobial exposure on the development of antimicrobial resistance (AMR) for macrolide antibiotics: protocol for a systematic review with a network meta-analysis
Source: Syst Rev. 2018 Dec 23;7:246. doi: 10.1186/s13643-018-0917-0 (PMC6304229; doi:10.1186/s13643-018-0917-0)
Supplement: Supplementary file 1 — Data extraction form. The draft form includes the risk of bias assessment tool and documents the data which will be extracted from included studies. (DOCX 26 kb) [file 13643_2018_917_MOESM1_ESM.docx]

# Additional file 1: Data extraction form. The draft form includes the risk of bias assessment tool and documents the data which will be extracted from included studies.

| Reviewer initials |  |
| --- | --- |
| Study ID |  |
| Review question | What is the relationship between duration of antimicrobial exposure and development of antimicrobial resistance (AMR)? |

## Table S1: Full text sift

| **Author** | |  |
| --- | --- | --- |
| **Citation** | |  |
| **Eligibility for review** | **Yes** |  |
|  | **No** |  |
| **Comments** - if study is not eligible, state reason(s) why | |  |

**Guide for completing Table S1**

| **Field** | **Content** |
| --- | --- |
| **Author** | Enter lead author last name e.g. Smith |
| **Citation** | State full study citation details |
| **Eligibility for review** | Refer to the latest version of the systematic review protocol for the eligibility criteria. |

## Table S2. Characteristics of included studies

| **Author** | |  |
| --- | --- | --- |
| **Year of publication** | |  |
| **Type of publication** | |  |
| **Funder** | |  |
| **Sponsor** | |  |
| **Conflicts of interest** | |  |
| **Country(ies) where conducted** | |  |
| **Study objectives** | Primary |  |
|  | Secondary |  |
|  | Further objectives |  |
| **Total study sample size** | |  |
| **Power calcuation performed?** | |  |
| **Methods of recruitment / selection** | |  |
| **Inclusion criteria** | |  |
| **Exclusion criteria** | |  |
| **Sequence Generation** | Random |  |
|  | Not random |  |
| **Allocation** | Blinded |  |
|  | Not blinded |  |
| **Selection bias and confounding variables** | |  |
| **Test for AMR ascertainment** | |  |
| **Timing of AMR ascertainment (days from prescription to test)** | |  |
| **Comments** | |  |

**Guide for completion of Table S2**

| **Field** | **Content** |
| --- | --- |
| Author | Enter lead author last name e.g. Smith |
| Year of publication | Enter year of publication |
| Type of publication | e.g. journal article, chapter, website, clinical guidelines, etc |
| Country(ies) where conducted | Enter the country/countries where the study was undertaken (if available) |
| Study objectives | List the objectives of the study (including secondary or tertiary as ‘further objectives’, where studied) |
| Study size | Enter the total sample size and whether any power calculation was carried out. (N.B. Where the study design was a cluster RCT, enter the number of clusters and total number of participants) |
| Methods of recruitment / selection | Enter the method of selection and recruitment of participants e.g. stratified random sampling from hospital inpatients, etc |
| Inclusion criteria | List any inclusion criteria used in selecting participants |
| Exclusion criteria | List any exclusion criteria used in selecting participants |
| Sequence generation | Indicate whether the the allocation sequence was randomly generated or not |
| Allocation | Indicate which group(s) were blinded to treatment allocation (or whether no-one was) |
| Selection bias and confounding variables | Enter any confounding variables stated in the text, methods used to measure them, and how these were controlled for by the study design |
| Test for AMR ascertainment | State the test used to determine AMR |
| Timing of AMR ascertainment | State number of days from antimicrobial prescription to AMR testing |
| Comments | Any further comments relevant to the extracted data |

## Table S3. Study population characteristics

| **Description of study arms** |  |
| --- | --- |
| **Description of eligibility criteria** |  |
| **Description and confirmation of target illness or syndrome** |  |
| **Sample size of study arm** |  |
| **Average age and measure of spread (eg variance/standard deviation)** |  |
| **Male %** |  |
| **Female %** |  |
| **Numbers recruited at each level of health care** |  |
| Primary care (n) |  |
| Hospital outpatient (n) |  |
| Hospital inpatient (n) |  |
| **Aetiology of infection identified (n)** |  |
| Prior antibiotics (yes/no) |  |
| Percentage of participants treated with prior antibiotics (%) within 30 days of enrollment |  |
| **Comments** |  |

**Guide for completion of Table S3**

| **Field** | **Content** |
| --- | --- |
| Description of study arms | Provide a brief description of each study arm e.g. cancer patients, splenectomised patients, healthy controls, etc |
| Description of eligibility criteria | Record inclusion and exclusion criteria of the study |
| Description and confirmation of target illness or syndrome | Provide details of how the target illness, leading to eligibility, was ascertained. |
| Sample size in study arm | Enter the number of patients included from each study arm |
| Setting | State setting e.g. acute hospital, out-patient clinic, GP surgery, residential care, community, etc |
| Average age and measure of spread | Enter the average (mean/median) age of participants in years, including measure of spread - report mean +/- standard deviation where possible |
| Gender | Indicate number of male and female participants |
| Care level | Provide numbers of participants recruited from each level of health care |
| Aetiology of infection identified (n) | Indicate number of cases where the aetiology of infection was isolated |
| Prior antibiotics | Did the study include any participants who used antibiotic prior to enrollment? |
| Percentage of participants treated with | Percentage reporting taking antibiotic within 30 days prior to enrollment |
| Comments | Comments relevant to the extracted data |

## Table S4. Study treatments

|  |  | **Intervention 1** | **Intervention 2** |
| --- | --- | --- | --- |
| **INTERVENTION** | Intervention (name the antimicrobial used) [use one line for each antimicrobial given serially] |  |  |
|  | Indication for the antimicrobial |  |  |
|  | n adults >18 in intervention arm |  |  |
|  |  |  |  |
|  | Duration of antimicrobial prescription (planned duration of prescription) |  |  |
|  | Timing of treatment assessment (number of days post-prescription) |  |  |
|  | Treatment compliance |  |  |
|  | Treatment dose |  |  |
|  | Source of drug (manufacturer versus local supplier) |  |  |
|  |  |  |  |
| **COMPARATOR** | Comparator arm (for inclusion need to be receiving no antimicrobial or alternative non-TB antimicrobial for the same indication as the intervention) |  |  |
|  | Indication for the antimicrobial |  |  |
|  | n adults >18 in comparator arm |  |  |
|  |  |  |  |
|  | Duration of antimicrobial prescription (planned duration of prescription) |  |  |
|  | Timing of treatment assessment (number of days post-prescription) |  |  |
|  | Treatment compliance |  |  |
|  | Treatment dose |  |  |
|  | Source of drug (manufacturer versus local supplier) |  |  |
| **Comments** | |  |  |

**Guide for completion of Table S4**

| **Field** | **Content** |
| --- | --- |
| Reviewer | Enter initials of person reviewing paper |
| Study ID | Enter unique ID |
| Care provider | State care provider e.g. hospital doctor, GP, nurse (hospital), nurse (community), etc |
| Intervention | For each antimicrobial, enter the details requested under this heading |
| Comparator | Enter the prescribed details for each comparator treatment |
| Comments | Any additional comments relevant to the extracted data |

## Table S5. Outcome measures

| **Outcome measure** | | **Primary outcome** | **Secondary outcomes** | | | |
| --- | --- | --- | --- | --- | --- | --- |
|  |  | Resistance in host | Symptom duration | Treatment failure | Disease recurrence | Resistance mechanism |
|  | Outcome definition from the article |  |  |  |  |  |
|  | Blinding |  |  |  |  |  |
| **Analysis** | Duration of follow-up |  |  |  |  |  |
|  | Intention to treat (ITT) or per protocol (PP) |  |  |  |  |  |
|  | Number included |  |  |  |  |  |
|  | Number included for measuring the outcome in the intervention arm |  |  |  |  |  |
|  | Number included for measuring the outcome in the comparator arm |  |  |  |  |  |
|  | Withdrawals - intervention |  |  |  |  |  |
|  | Withdrawals - comparator |  |  |  |  |  |
|  | Exclusions - intervention |  |  |  |  |  |
|  | Exclusions - comparator |  |  |  |  |  |
|  | Loss to follow up - intervention |  |  |  |  |  |
|  | Loss to follow up - comparator |  |  |  |  |  |
|  | Result - intervention |  |  |  |  |  |
|  | Result - comparator |  |  |  |  |  |
|  | Result - statistical output |  |  |  |  |  |
|  | Control for selection bias and confounding |  |  |  |  |  |
|  | Are RCT arms balanced based on the basis of baseline characteristics? |  |  |  |  |  |
| **Comments** | |  |  |  |  |  |

**Guide for completion of Table S5**

| **Field** | **Content** |
| --- | --- |
| Outcome |  |
| - definition | Enter the definition of each outcome measure considered |
| - blinding | Indicate which group(s) were blind to allocation or whether no-one was |
| Analysis |  |
| - duration of follow-up | Enter the length of time over which outcome measurements were made |
| - method | Enter the method of statistical analysis employed |
| - ITT / PP | Enter if the analysis conducted was intention to treat or per protocol |
| - number included | Enter the number of measurements included in the analysis |
| Numbers included for measuring the outcome in the intervention arm | Enter the number of participants included in the evaluations of outcomes in the intervention group. |
| Numbers included for measuring the outcome in the comparator arm | Enter the number of participants included in the evaluations of outcomes in the comparator group. |
| - withdrawals - intervention | Enter the number of withdrawals from the allocated intervention group |
| - withdrawals - comparator | Enter the number of withdrawals from the allocated comparator group |
| - exclusions - intervention | Enter the number of exclusions from the allocated intervention group |
| - exclusions - comparator | Enter the number of exclusions from the allocated comparator group |
| - loss to follow up - intervention | Enter the number of participants lost to follow up from the intervention group |
| - loss to follow up - comparator | Enter the number of participants lost to follow up from the comparator group |
| - Result - intervention | Enter the intervention result as expressed in the article (e.g. mean +/- standard deviation, number of cases with outcome vs all cases, etc; include baseline and follow-up data points) |
| - Result - comparator | Enter the intervention result as expressed in the article (e.g. mean +/- standard deviation, number of cases with outcome vs all cases, etc - include baseline and follow-up data points) |
| - Result - statistical output | Enter full details of the statistical test output including estimate of precision (e.g. 95% confidence interval), test statistic and p value - include adjusted odds ratios where available |
| - control for selection bias and confounding | Enter 'yes', 'no' or 'not applicable' and provide brief details of how selection bias and confounding variables were or were not controlled for during the statistical analysis |
